# Clinical Outcome of Day-3 Cleavage Slow-Growing Embryos at Different Cleavage Rates after Overnight Culture: A Cohort Retrospective Study

**DOI:** 10.3390/jcm11154417

**Published:** 2022-07-29

**Authors:** Lan Geng, Xinran Lin, Rang Liu, Jiahui Wu, Yongsheng Luo, Hongmei Sun, Zhenhui Hou, Qiuju Zhang, Chang Xu, Xiao Li, Canhui Cao, Tianren Wang, Xi Xia

**Affiliations:** 1 Center for Reproductive Medicine, Peking University Shenzhen Hospital, Shenzhen 518000, China; genglan9@163.com (L.G.); 16rliu2@stu.edu.cn (R.L.); shm863@sina.com (H.S.); houstarry@sina.com (Z.H.); mzzqj@126.com (Q.Z.); wangchang0421@sina.com (C.X.); canhuicao@foxmail.com (C.C.); 2 Medical College, Shantou University Medical College, Shantou 515063, China; Xr_Lin1@163.com; 3 Department of Gynecology, Shenzhen Maternity & Child Healthcare Hospital, Shenzhen 518000, China; jiahui_313@163.com; 4 Quality Control Department, The Second People’s Hospital of Futian District, Shenzhen 518000, China; Luozi99167@126.com; 5 Shenzhen Health Capacity Building and Continuing Education Center, Shenzhen 518000, China; lix@wjw.sz.gov.cn; 6 Center for Reproductive Medicine, The University of Hong Kong-Shenzhen Hospital, Shenzhen 518000, China

**Keywords:** slow-growing embryo, overnight culture, frozen embryo transfer, cleavage rate

## Abstract

Introduction: We explored the association between clinical outcomes and the cleavage rate of day-3 cleavage slow-growing embryos after overnight culture. Methods: The data collected from 303 frozen embryo transfer (FET) cycles with 606 4-cell or 5-cell embryos cultured overnight (18–22 h) after thawing were analyzed. Based on the growth rate after the overnight culture, the embryos were divided into three groups: no embryo reaching eight cells (Group I), either one of the two embryos reaching eight cells (Group II), and both two embryos reaching eight cells or more (Group III). A statistical analysis of the different clinical outcomes from the three groups was performed. Results: Biochemical pregnancy rate (OR 3.22; *p* = 0.001), implantation rate (OR 2.44; *p* = 0.002), clinical pregnancy rate (OR 3.04; *p* = 0.001), ongoing pregnancy rate (OR 3.14; *p* = 0.001), and live birth rate (OR 2.78; *p* = 0.004) were significantly higher in Group III as compared to Group I. Group II had a significantly higher biochemical pregnancy rate (OR 2.02; *p* = 0.013) and implantation rate (OR 1.77; *p* = 0.019) than Group I. Conclusions: The capability of day-3 cleavage slow-growing embryos to reach eight cells, especially that of two embryos reaching eight cells by overnight culture, appear to result in a better pregnancy outcome.

## 1. Introduction

The developmental potential of embryos is fundamental to the outcomes of assisted reproductive technology. Embryo quality, including the cellular number and other morphological parameters, are generally considered to be the crucial indicators of developmental potential [1]. Previous studies have suggested that day-3 slow-growing embryos that have less than six cells have a comparatively low implantation rate and live birth rate [2,3,4,5,6]. The ASEBIR consensus scheme suggested in 2011 that day-3 slow-growing embryos cloud not be recommended to be transferred [1]. Recently, the developmental potential of slow-growing embryos by further culturing has been investigated by several studies. For instance, Heather Burks suggested that implantation rates with the use of embryos reaching eight cells on day 4 and the embryos reaching eight cells on day 3 were not significantly different in the frozen embryo transfer (FET) cycles [7]. Some researchers extended the culture of slow-growing embryos until day 5 and suggested that the slow-growing embryos can develop to good-quality blastocysts, although the ratio is relatively low [8,9]. Studies have also suggested that slow-growing embryos had development potential and should be cultured for more time to evaluate their potential. Until now, information on how to effectively evaluate the developmental potential of slow-growing embryos is lacking.

Overnight culture is an alternative method for FET in which further embryo division could be observed. J.Van der Elst and Elia Fernandez Gallardo et al. showed that a higher implantation rate was obtained from embryos that further cleaved after being cultured overnight [10,11]. Several other studies also focused on the pregnancy outcomes of developing embryos after overnight culture. Compared with embryos developing more slowly, a higher pregnancy rate could be observed in faster-developing counterparts, which indicated that cleavage rate was a good predictor of clinical outcomes after overnight culture [12,13]. However, surveys devoted to the overnight culture of slow-growing embryos are still lacking.

Whether the cleavage rate could indicate the embryo potential and be associated with the clinical outcome of slow-growing, day-3 embryos are unclear. Therefore, the present study aimed to explore the association between clinical outcomes and cleavage rate of day-3 cleavage slow-growing embryos after overnight culture.

## 2. Materials and Methods

### 2.1. Participant

**Figure 1:**
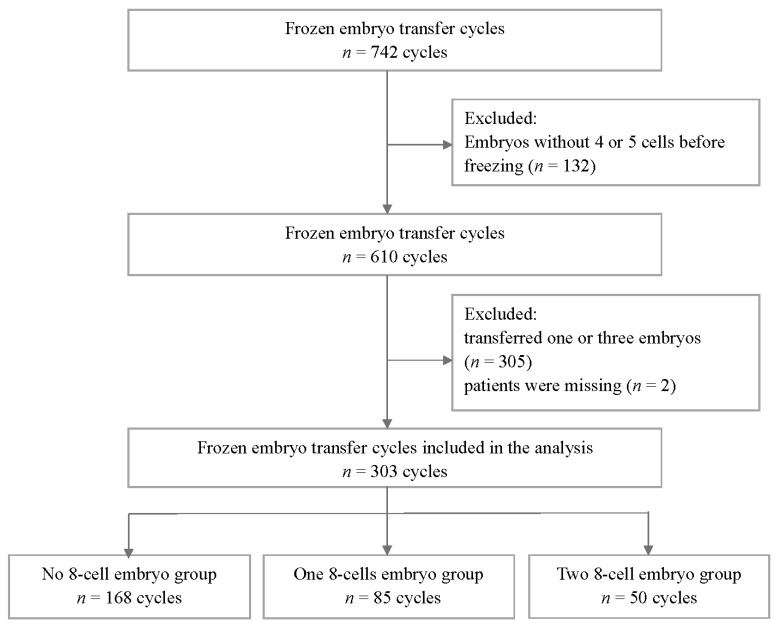
Flow diagram showing the distribution of the study populations.

The retrospective cohort study was performed at the Reproductive Medicine Center in Peking University Shenzhen Hospital. A total of 742 FET cycles conducted between January 2017 and September 2019 were analyzed, and 610 FET cycles in which day-3 embryos with 4 or 5 cells that had undergone overnight culture were selected. A total of 307 cycles with one or three transferred embryos and with incomplete records were excluded. Finally, 606 cleavage embryos that were cultured overnight (18–22 h) after thawing were divided into three groups according to their cleavage rate: no embryo reaching 8 cells (Group I), either one of the two embryos reaching 8 cells (Group II), and both two embryos at least reaching 8 cells (Group III). Figure 1 shows the protocol for this study.

### 2.2. Ovarian Stimulation, Oocyte Retrieval, and Fertilization

The protocol for controlled ovarian hyperstimulation was based on personal characteristics. Transvaginal ultrasonography and measurements of serum estradiol (E2) levels were used to monitor follicular growth. When at least two follicles reached a mean diameter of 18 mm, a dose of 6000 IU of human chorionic gonadotropin (hCG; Choriomon, IBSA, Lugano, Switzerland) was administered intramuscularly. Oocyte retrieval was performed 36 h after hCG injection. Regular in vitro fertilization (IVF) or intracytoplasmic sperm injection (ICSI) was performed according to the patients’ indications. Fertilization assessment was performed 16–18 h after insemination or injection to check for the appearance of two distinct pro-nuclei and two polar bodies.

### 2.3. Embryo Assessment, Freezing, and Thawing

According to the routine evaluation system, the morphology of embryos was evaluated for the cell number, fragmentation, and symmetry on day 3 after insemination [1]. On day 3, embryos with 4 or 5 blastomeres and ≤25% fragmentation were selected for cryopreservation. The vitrification and warming procedures were performed according to standard protocols of vitrification and the warming kits (Kitazato, Shizuoka, Japan).

For vitrification, the embryos were firstly transferred into the equilibration solution for 9 min and then placed into the vitrification solution for 1 min. Next, the embryos were placed in a cryotron and put into the liquid nitrogen within the same device for 60 s. For warming, the embryos were taken out of the liquid nitrogen and immediately placed into a thawing solution for 1 min. Subsequently, the embryos were plunged into a dilution solution at room temperature for 5 min, followed by washing solution 1 for 5 min, and then washing solution 2 for 1 min, before finally being transferred into the culture medium. Embryos with at least 50% of their cells intact were considered to be surviving and were cultured overnight for 18–22 h. Further cleavage was evaluated the next morning, and the cell number was counted.

### 2.4. Endometrium Preparation and Embryos Transfer

The embryos were transferred either in natural or hormonally supplemented cycles. As for the natural cycle, the method of ultrasound examination was adopted to observe follicular growth. Once the diameter of the follicle was ≥18 mm, and while the endometrium thickness was ≥7 mm, 5000–10,000 IU urinary hCG (Choriomon, IBSA, Lugano, Switzerland) was administered to trigger ovulation. To achieve endometrium preparation for the hormone replacement therapy cycle, oral estradiol valerate (Progynova, Bayer-Schering Pharma AG, Berlin, Germany confirms) was given to the patients. While the E2 and endometrial thickness were suitable, P supplementation was started. After an overnight culture, the embryo was then transferred.

### 2.5. Main Outcome Measures

If the Beta-Human Chorionic Gonadotrophin (βhCG) level in the blood on the 14th day after embryo transfer was higher than 50 mIU/mL, it was considered a biochemical pregnancy. The implantation rate (IR) was the number of observed gestational sacs per number of thawed embryos transferred. Clinical pregnancy was affirmed if gestational sacs could be observed by ultrasound 5 weeks after embryo transfer. Ongoing pregnancy was confirmed by the detection of the fetal heartbeat during the 12-week ultrasound examinations. Miscarriage rate (MR) was calculated as the number of clinical pregnancy lost cycles divided by clinical pregnancy cycles. Multiple pregnancy rate (MPR) refers to the number of multiple pregnancy cycles divided by the clinical pregnancy cycles. Live birth rate (LBR) was defined as the number of live deliveries per FET cycle.

### 2.6. Statistical Analysis

The data were analyzed by SPSS 20.0. Statistical significance was defined as *p* < 0.05. The continuous data, including female age at FET treatment, body mass index (BMI), basal follicle-simulating hormone (FSH), basal estrogen (E2), anti-Müllerian hormone (AMH), the endometrial thickness, and the number of oocytes retrieved were analyzed by one-way analysis of variance (ANOVA) and presented as the mean ± standard deviation (SD). As for categorical data, the chi-square test or Fisher’s exact test was adopted for the comparison of the factor of infertility, endometrium preparation, biochemical pregnancy rate, clinical pregnancy rate, ongoing pregnancy rate, implantation rate, multiple pregnancy rate, miscarriage rate, and live birth rate. The proliferation cycles were compared using the Kruskal–Wallis test. Logistic regression analysis was used to adjust for confounders, including age, BMI, AMH, and endometrial thickness. According to the Bonferroni adjustment for multiple comparisons, *p* < 0.0167 (calculated as 0.05/3) was considered statistically significant among the three groups.

## 3. Results

According to the cell number of the overnight-cultured embryos, the embryos were categorized into a non-eight-cell group (Group I, *n* = 168), a one eight-cell group (Group II, *n* = 85), and a two eight-cell group (Group III, *n* = 50).

As shown in Table 1, the background characteristics, including age, BMI, factors of infertility, endometrium preparation, basal FSH, basal E2, and AMH were not significantly different among three groups (*p* > 0.05).

Table 2 presents the laboratory characteristics of embryos after overnight culture. In total, 606 embryos were observed: 336 embryos in Group I, 160 embryos in Group II, and 100 embryos in Group III. There was no significant difference in the number of oocytes retrieved from the three groups.

As presented in Table 3, the thickness of the endometrium before the transfer did not differ when compared among the three groups (*p* > 0.05). The biochemical pregnancy rate (BPR) of Group II (43.5%) was higher than that of Group I (28.0%) (*p* = 0.016). As for the IR, clinical pregnancy rate (CPR), ongoing pregnancy rate (OPR), MPR, MR, and LBR between Group II and Group I, no statistical significance could be found (*p* > 0.0167). The BR, IR, CPR, OPR, and LBR significantly differed between Group III and Group I (*p* < 0.0167), while the MPR and MR showed no significant differences.

Table 4 shows the results of the logistic regression analysis. To eliminate the effects on clinical outcomes, several variables, including age, BMI, AMH, and endometrial thickness, were employed in the logistic regression analysis. After adjustment for confounders, Group III was shown to have higher BPR (OR 3.22; 95%; CL 1.65–6.26; *p* = 0.001), IR (OR 2.44; 95% CL 1.42–2.40; *p* = 0.001), CPR (OR 3.04; 95% CL 1.56–5.92; *p* = 0.001), OPR (OR 3.14; 95% CL 1.59–6.21; *p* = 0.001), and LBR (OR 2.78; 95% CL 1.40–5.53; *p* = 0.004) than Group I. Group II has a significantly higher BPR (OR 2.02; 95% CL 1.16–3.53; *p* = 0.013) and IR (OR 1.77; 95% CL 1.10–2.86; *p* = 0.019) than Group I.

## 4. Discussion

To our knowledge, the developmental potential of day-3 slow-growing embryos after overnight culture and their related clinical outcomes in frozen–thawed cycles have never been systematically described. The aim of this retrospective analysis was to clarify the association of clinical outcomes with the cleavage rate of the day-3 slow-growing embryos after overnight culture. As shown in the present study, better clinical outcomes could be observed in groups where embryos could reach eight cells, especially in the group in which two embryos reached eight cells, indicating that the cleavage rate after overnight culture may serve as a positive prognostic factor for the IVF outcomes of these slow-growing embryos.

We found that Nigel Pereira’s study was also interested in the growth of 4-cell embryos. They reported that by culturing from morning until afternoon, the growth group had a higher CPR (13.9% vs. 4.49%) and LBR (10.9% vs. 3.37%) as compared to the group that continued to be 4-cell embryos [14]. Notably, the incidences of BPR, IR, and MR were not reported in the aforementioned study. Moreover, the grouping method applied in the literature was too simple. In addition, previous studies have been conducted on fresh embryo transfer cycles. The present study, which paid more attention to the growth rate of delayed embryos after overnight culture, clearly demonstrated that growth rate could be served as a positive prognostic factor of the clinical outcome.

In accordance with our present results, previous studies on the general embryo population demonstrated that the transfer of embryos that had cleaved during overnight culture resulted in a significantly higher CPR than the transfer of those without any cleavage [12]. Embryos with further cleavage always seemed to be a positive prognostic factor of embryo developmental potential, especially once the embryos reached eight cells [7]. Before freezing, when the slow-growing embryos may not have undergone embryonic genome activation (EGA) yet—which the study showed to occur between the four-cell and eight-cell stages—human gene expression first occurs. Embryos with developmental potential have the opportunity to develop to more than eight cells by overnight culture because of the EGA [15]. It is believed that overnight culture, which provides the embryos with a chance to “catch up” and allows for the occurrence of EGA, is beneficial to the embryos with developmental potential, especially for the embryos that were slow-growing on day 3. Embryos that had not yet undergone the EGA during overnight culture resulted in a slower cleavage rate, which would possibly lead to poor clinical outcomes.

As reported by Heather Burks et al. in their study, although the embryos did not reach eight cells until day 4, their associated CPR did not differ from those of the day-3 eight-cell embryos after adjusting for possible confounders [7]. It is worth mentioning that Group III in our study, in which two day-3 slow-growing embryos reached eight cells after overnight culture, had a higher clinical pregnancy rate as compared with the values in the study of Heather Burks et al. The difference between the clinical pregnancy rates in the two studies may be explained mainly by age. While the mean age of the targeted population in the study of Heather Burks et al. was 39, the corresponding age in our study was 33.7. It was demonstrated that as age increased, mitochondrial function deteriorated, thus negatively impacting embryo competence [16]. In our study, transferring day-3 slow-growing embryos led to a 36.5% clinical pregnancy rate for Group II and 50.0% for Group III, and ultimately resulted in a 30.6% live birth rate for Group II and 40.0% for Group III. As reported, the clinical pregnancy rate per thawing cycle for FET in mainland China in 2016 was 48.2% and the delivery rate was 37.6% [17]. Considering the rather acceptable results in our study and the findings in the previous study [7], it is speculated that overnight culture may be an alternative strategy when dealing with day-3 slow-growing embryos, and this kind of embryos could be taken into consideration for younger women to try in their cycle when no embryos of better quality are available.

With the development of technology, the transfer of blastocyst instead of cleavage-stage embryos has become an increasing trend in ART. The advantage of transferring blastocyst is that it improves both uterine and embryonic synchronicity and enables embryo self-selection after the activation of the embryonic genome [18,19]. However, the culture system may lead to the risk of losing a part of the embryos, which may not survive the challenge of extended culture [20]. According to previous studies, the blastulation rate that originated from slow-growing embryos was between 20% and 50% [21,22,23], which may reflect the fact that at least one of the halves of the slow-growing embryos may fail to extend to blastocysts. On the one hand, the day-3 slow-growing embryos decrease the chances of achieving the blastocyst stage [24]; on the other hand, the culture system also plays an important role that may have a negative effect on embryos. The discussions on the clinical outcomes of blastocysts derived from the slow-growing embryos have remained contradictory. Previous studies have reported that transferring blastocysts derived from slow-growing embryos significantly influenced clinical pregnancy outcomes [9,22], while other studies showed that similar clinical outcomes could be observed once the slow-growing embryos extended to the blastocysts stage [6,25,26]. Therefore, culturing all the slow-growing embryos to the blastocyst stage may not be suitable for each patient. Our study shows that the embryos of Group III should be transferred or that culturing to blastocysts should be further studied. As for Group I, the embryos may be cultured into blastocysts to reduce the transfer cycle. Our study may offer a new way of thinking to maximize the use of slow-growing embryos, which may reduce treatment expenses and ease the pressure from intervention procedures for some patients.

The synchrony between embryo maturation and endometrial development, which are two independent events, is a crucial factor for successful implantation. It was reported that a lower implantation rate may result when there is a time difference between the slow growth of the embryo and the accelerated endometrium decidualization [27,28]. In the current study, further overnight culture once the two embryos reach eight cells may correct the synchrony between the embryo and the endometrium, and thus result in relatively satisfactory pregnancy outcomes.

There are some limitations to this study. Firstly, it is a retrospective cohort study, limiting the strength of the evidence for the current conclusion. High-quality RCTs are needed for further investigation. Secondly, we paid more attention to the cell number than the fragmentation, although Elia Fernandez Gallardo et al. showed that the clinical outcomes were determined by the occurrence of mitosis resumption and the specific number of blastomeres, instead of the fragmentation, blastomere symmetry, or volume change [11].

## 5. Conclusions

Culturing the slow-growing, day-3 embryos overnight and transferring those at a faster cleavage rate can result in an improvement in the clinical outcomes of frozen embryo transfer. The growth rate of the slow-growing embryos by overnight culture could indicate more successful clinical outcomes and may provide some references for clinical decision making when dealing with slow-growing embryos.

## Figures and Tables

**Table 1:** Demographic and clinical characteristics of the three groups.

	Group I	Group II	Group III	*p*
No. of cycles	168	85	50	
Maternal age (years)	32.6 ± 3.6	32.9 ± 3.6	33.7 ± 4.7	0.183
BMI (kg/m^2^)	20.8 ± 2.9	20.5 ± 2.5	21.3 ± 2.6	0.377
Factors of infertility (%)				0.414
Female	91 (54.2)	47 (55.3)	26 (52.0)	
Male	50 (29.8)	18 (21.2)	17(34.0)	
Both	10 (6.0)	4 (4.7)	2 (4.0)	
Unexplained	17 (10.1)	16 (18.8)	5 (10.0)	
Endometrial preparation (%)				0.127
NC	88 (52.4)	41 (48.2)	18 (36.0)	
HRT	80 (47.6)	44 (51.8)	32(64.0)	
Basal FSH (IU/L)	9.2 ± 3.0	7.7 ± 2.8	7.6 ± 2.9	0.288
Basal E2 (IU/L)	55.1 ± 49.2	60.6 ± 54.5	55.5 ± 26.5	0.406
AMH (ng/mL)	4.8 ± 3.6	4.8 ± 3.7	4.4 ± 3.8	0.734

Values are reported as means ± standard deviations or numbers (percentages).

**Table 2:** The laboratory characteristics of thawed embryos following overnight culture.

	Group I	Group II	Group III	*p*	*p*-Value ^a^	*p*-Value ^b^	*p*-Value ^c^
	(*n* = 168)	(*n* = 85)	(*n* = 50)				
No. of post-thawed embryos	336	170	100				
No. of oocytes retrieved	11.8 ± 5.9	11.4 ± 5.2	10.6 ± 6.0	0.394	0.591	0.176	0.411
Conventional IVF (%)	96 (57.1)	51 (60.0)	30 (60.0)	0.875	0.729	0.863	1.000
Proliferation cycles ^d^				<0.001	<0.001	<0.001	<0.001
Without further	42(25.0)	0	0				
development cycles							
One for further	69 (41.1)	25 (29.4)	0				
development cycles							
Two for further	57 (33.9)	60 (70.6)	50 (100)				
development cycles							

Values are reported as means ± standard deviations, oras numbers (percentages). The Kruskal–Wallis test was used to investigate proliferation capacity among the three groups. ^a^ Group II vs. Group I. ^b^ Group III vs. Group I. ^c^ Group III vs. Group II. ^d^ Proliferation cycles were defined as the number of cycles in which the amount of embryo cells increased.

**Table 3:** Clinical outcomes of the three groups.

	Group I	Group II	Group III	*p*	*p*-Value ^a^	*p*-Value ^b^	*p*-Value ^c^
	(*n* = 168)	(*n* = 85)	(*n* = 50)	*p* < 0.05	*p* < 0.0167		
Endometrial	11.8 ± 2.3	11.4 ± 2.2	11.1 ± 2.1	0.121	0.243	0.051	0.371
thickness (mm)							
Biochemical pregnancy	47/168 (28.0)	37/85 (43.5)	27/50 (54.0)	0.001	0.016	0.001	0.285
rate (%)							
Implantation	48/336 (14.3)	38/170 (22.4)	28/100 (28.0)	0.003	0.025	0.002	0.038
rate (%)							
Clinical pregnancy	43/168 (25.6)	31/85 (36.5)	25/50 (50.0)	0.004	0.080	0.002	0.149
rate (%)							
Ongoing pregnancy	37/168 (22.0)	28/85 (32.9)	23/50 (46.0)	0.004	0.068	0.001	0.145
rate (%)							
Multiple pregnancy	5/43 (11.6)	7/31 (22.6)	3/25 (12.0)	1.000	1.000	1.000	1.000
rate (%)							
Miscarriage	6/43 (14.0)	3/31 (9.7)	4/25 (16.0)	0.806	0.726	1.000	0.688
rate (%)							
Live birth	36/168 (21.4)	26/85 (30.6)	21/50 (42.0)	0.012	0.123	0.006	0.195
rate (%)							

The chi-square test or Fisher’s exact test was used to compare the three groups. ^a^ Group II vs. Group I. ^b^ Group III vs. Group I. ^c^ Group III vs. Group II.

**Table 4:** Logistic regression analysis: the relationship between the groups and clinical outcomes.

	Group II vs. Group I		Group III vs. Group I		Group III vs. Group II	
	OR (95%CI)	*p*-Value	OR (95%CI)	*p*-Value	OR (95%CI)	*p*-Value
Biochemical pregnancy rate ^a^						
Unadjusted	1.98 (1.15–3.42)	0.014	3.02 (1.57–4.75)	0.001	1.59 (0.78–3.26)	0.240
Adjusted	2.02 (1.16–3.53)	0.013	3.22 (1.65–6.26)	0.001	1.53 (0.75–3.08)	0.204
Implantation rate ^a^						
Unadjusted	1.73 (1.08–2.77)	0.023	2.33 (1.37–3.98)	0.002	1.35 (0.77–2.38)	0.298
Adjusted	1.77 (1.10–2.86)	0.019	2.44 (1.42–2.40)	0.001	1.38 (0.78–2.45)	0.275
Clinical pregnancy rate ^a^						
Unadjusted	1.67 (0.95–2.93)	0.074	2.91 (1.51–5.59)	0.001	1.74 (0.86–3.54)	0.125
Adjusted	1.70 (0.96–3.00)	0.069	3.04 (1.56–5.92)	0.001	1.79 (0.87–3.68)	0.113
Ongoing pregnancy rate ^a^						
Unadjusted	1.74(0.97–3.11)	0.062	3.02 (1.55–5.87)	0.001	1.73 (0.85–3.55)	0.132
Adjusted	1.72 (0.95–3.11)	0.072	3.14 (1.59–6.21)	0.001	1.83 (0.88–3.80)	0.105
Multiple pregnancy rate ^a^						
Unadjusted	1.13 (0.28–4.59)	0.869	1.04(0.23–4.76)	0.963	0.92(1.87–4.56)	0.919
Adjusted	1.30(0.28–6.03)	0.739	0.97 (0.19–4.90)	0.969	0.75 (0.14–4.13)	0.737
Miscarriage rate ^a^						
Unadjusted	0.66 (0.15–2.87)	0.581	1.18 (0.30–4.64)	0.818	1.64 (0.30–8.86)	0.481
Adjusted	0.70 (1.43–3.46)	0.665	1.15 0.27–4.96)	0.851	1.78 (0.36–8.82)	0.568
Live birth rate ^a^						
Unadjusted	1.62 (0.90–2.92)	0.111	2.66 (1.36–5.20)	0.004	1.64 (0.79–3.40)	0.180
Adjusted	1.63 (0.89–2.96)	0.112	2.78 (1.40–5.53)	0.004	1.71 (0.82–3.59)	0.156

^a^ After adjusting for age, BMI, AMH, and endometrial thickness. OR, odds ratio; 95%CI, 95% confidence interval.

## Data Availability

The datasets analyzed during the current study are available from the corresponding author on reasonable request.

## Abbreviations

FET: Frozen embryo transfer; IVF: In vitro fertilization; ICSI: Intracytoplasmic sperm injection; BMI: Body Mass Index; FSH: Follicle-simulating hormone; AMH: Anti-Müllerian; E2: Estrogen; IR: Implantation Rate; MR: Miscarriage Rate; MPR: Multiple Pregnancy Rate; LBR: Live Birth Rate; BPR: Biochemical Pregnancy Rate; CPR: Clinical Pregnancy Rate; OPR: Ongoing Pregnancy Rate.
